# Kinetics of Neutralizing Antibodies of COVID-19 Patients Tested Using Clinical D614G, B.1.1.7, and B 1.351 Isolates in Microneutralization Assays

**DOI:** 10.3390/v13060996

**Published:** 2021-05-26

**Authors:** Jenni Virtanen, Ruut Uusitalo, Essi M. Korhonen, Kirsi Aaltonen, Teemu Smura, Suvi Kuivanen, Sari H. Pakkanen, Sointu Mero, Anu Patjas, Marianna Riekkinen, Anu Kantele, Visa Nurmi, Klaus Hedman, Jussi Hepojoki, Tarja Sironen, Eili Huhtamo, Olli Vapalahti

**Affiliations:** 1 Department of Virology, Faculty of Medicine, University of Helsinki, 00290 Helsinki, Finland; jenni.me.virtanen@helsinki.fi (J.V.); ruut.uusitalo@helsinki.fi (R.U.); kirsi.aaltonen@helsinki.fi (K.A.); teemu.smura@helsinki.fi (T.S.); suvi.kuivanen@helsinki.fi (S.K.); visa.nurmi@helsinki.fi (V.N.); klaus.hedman@helsinki.fi (K.H.); jussi.hepojoki@helsinki.fi (J.H.); tarja.sironen@helsinki.fi (T.S.); eili.huhtamo@helsinki.fi (E.H.); olli.vapalahti@helsinki.fi (O.V.); 2 Department of Veterinary Biosciences, Faculty of Veterinary Medicine, University of Helsinki, 00790 Helsinki, Finland; 3 Department of Geosciences and Geography, Faculty of Science, University of Helsinki, 00560 Helsinki, Finland; 4 Human Microbiome Research Program, Faculty of Medicine, University of Helsinki, 00014 Helsinki, Finland; sari.pakkanen@hus.fi (S.H.P.); sointu.mero@helsinki.fi (S.M.); anu.patjas@helsinki.fi (A.P.); marianna.riekkinen@helsinki.fi (M.R.); anu.kantele@helsinki.fi (A.K.); 5 Meilahti Infectious Diseases and Vaccine Research Center (MeiVac), Inflammation Center, Helsinki University Hospital and University of Helsinki, 00290 Helsinki, Finland; 6 Virology and Immunology, Diagnostic Center, HUSLAB, Helsinki University Hospital, 00260 Helsinki, Finland; 7 Vetsuisse Faculty, Institute of Veterinary Pathology, University of Zürich, 8006 Zürich, Switzerland

**Keywords:** SARS-CoV-2, neutralizing antibodies, immunity

## Abstract

Increasing evidence suggests that some newly emerged SARS-CoV-2 variants of concern (VoCs) resist neutralization by antibodies elicited by the early-pandemic wild-type virus. We applied neutralization tests to paired recoveree sera (*n* = 38) using clinical isolates representing the first wave (D614G), VoC1, and VoC2 lineages (B.1.1.7 and B 1.351). Neutralizing antibodies inhibited contemporary and VoC1 lineages, whereas inhibition of VoC2 was reduced 8-fold, with 50% of sera failing to show neutralization. These results provide evidence for the increased potential of VoC2 to reinfect previously SARS-CoV-infected individuals. The kinetics of NAbs in different patients showed similar decline against all variants, with generally low initial anti-B.1.351 responses becoming undetectable, but with anti-B.1.1.7 NAbs remaining detectable (>20) for months after acute infection.

## 1. Introduction

Neutralizing antibodies (NAbs), most of which target the receptor binding domain (RBD) of viral spike protein, protect against SARS-CoV-2 infection [1,2,3,4,5,6]. However, some of the recently emerged variant SARS-CoV-2 lineages have raised concerns with regard to lowered protective immunity in vaccinees and recoverees due to mutations that cause neutralization escape [7,8,9,10]. Studies have indicated that the B.1.1.7-variant is neutralized almost equally to the original virus type, whereas increasing evidence suggests that the B.1.351 variant is less well neutralized.

Currently, no “golden standard” methodology exists for measuring NAbs against SARS-CoV-2, and assays have been performed with a variety of techniques such as SARS-CoV-2 spike-variant pseudotyped lentivirus [11,12,13], VSV [14,15,16], infectious clones, and virus isolates [17,18]. Cell lines have also varied, with some expressing transmembrane protease serine 2 (TMPRSS2) and/or angiotensin-converting enzyme 2 (ACE2), known to mediate SARS-CoV-2 entry. SARS-CoV-2 is known to readily adapt to Vero E6 (VE6) cells that have low TMPRSS2 but high ACE2 expression [19]. This can result in deletions around the furin cleavage site and force the viral entry to occur mainly via the endosomal route aided by alternate proteases.

In order to estimate to which extent and for how long antibodies raised by earlier infection protect against new variants of concern (VoC), and for optimizing the routine microneutralization tests (MNT) in use, we tested a panel of 38 paired sera from 18 laboratory-confirmed COVID-19 patients from spring and summer 2020 in MNT with different clinical isolates of SARS-CoV-2 including the B.1.1.7 and B 1.351 strains (now prevalent in the country) using Vero E6 (VE6) cells with and without expression of TMPRSS2.

## 2. Materials and Methods

### 2.1. Patient Samples 

The samples included 38 sera from 18 laboratory-confirmed patients from spring and summer 2020 drawn 2–4 weeks after the disease and 2–8 months later (Table S1). Patients had either been recovering at home or were treated in the hospital in regular wards or in intensive care units (ICUs). The study was performed according to research and ethical permit of HUS (detailed in Institutional Review Board Statement), and informed consent was obtained from all patients. 

### 2.2. Cell Lines 

Vero E6 cells (VE6) and their TMRPRSS2-expressing clone VE6-TMPRSS2-H10 [20] (VE6T) were grown in minimal essential eagle’s medium (MEM, Sigma-Aldrich, Saint Louis, USA) including 10% (cell maintenance) or 2% (infection experiments) fetal bovine serum (FBS, ThermoFisher, Waltham, USA), 2 mM L-glutamine, 100 IU/mL penicillin, and 100 µg/mL streptomycin.

### 2.3. Virus Strains

Tests were performed with 4 clinical virus isolates: SARS-CoV-2/Finland/1/2020 (FIN-1), C1P1, VoC1, and VoC2. FIN-1 (passage 7) is a VE6 passaged virus strain that was isolated from the first patient in Finland in January 2020 [21]. C1P1 is a wild-type low-passage strain representing strains circulating in Finland during spring 2020 (with e.g., the D614G mutation), devoid of mutations around the furin-cleavage site [19]. Strains representing typical B.1.1.7 (VoC1) and B.1.351 (VoC2) strains (Table S2) were isolated in VE6T cells from patient nasopharyngeal samples (200 µL) as previously described [20] and used as low-passage (p1 and p0) stocks. Virus replication was determined by RdRp-targeting RT-PCR [22]. The infectious virus titers were determined by plaque assay in VE6T cells, and the isolates were sequenced as previously described (Table S2) [19].

### 2.4. Comparing Microneutralization Tests with Different Cell Lines and Virus Strains 

Microneutralization tests were performed in a BSL3 level laboratory following the protocol published by Haveri et al. [21]. To address the host cell line effects with regard to neutralization, patient NAb titers from 1:20 serum dilution onwards were first compared in triplicate reactions both in VE6 cells and VE6T cells with the FIN-1 strain. VE6-TMPRSS2-H10 cells were also tested with C1P1-strain. Next, NAb titers against wild-type C1P1 and variant stains VoC1 and VoC2 were determined and compared using VE6T cells. 

### 2.5. Determining Anti-NP- and Anti-Spike-Titers

Anti-Spike-IgG and anti-nucleoprotein-IgG titers were determined by ELISA against respective antigens based on the first strains reported from Wuhan in January 2020, as described previously [17,23,24].

### 2.6. Statistical Testing

Statistical tests were performed with IBM SPSS Statistics 25. Titers of <20 were set to 10 for calculations. Values 1, 5, and LOD/$\sqrt{2}$ were also tested for titers <20, but those did not change the significance levels (0.05, 0.01, and 0.001) of statistical tests between virus strains. Due to the data not being normally distributed, non-parametric related-samples Wilcoxon signed rank test and related-samples Friedman’s two-way analysis of variance by ranks tests were used for testing the significances of the differences between virus strains, and the non-parametric Mann–Whitney U test was used to test the differences between subgroups (0–150 days and over 150 days from the onset of symptoms, and whether the patient was treated at home or at hospital). To test the differences in NAb titer decrease rate between C1P1, VoC1, and VoC2, slopes of fit lines between paired samples of individual patients were calculated and compared with Friedman’s two-way analysis of variance by ranks test. Log2 transformed titers were used in the y-axis and days from the onset of the disease in the x-axis. Only samples with the determinable titer were used in this analysis. 

## 3. Results

### 3.1. Optimizing MNT Protocol 

We first wanted to ensure that the titers obtained reflected neutralization against circulating wild-type strains in cells with relevant entry molecules. Hence, we compared neutralizing antibody titers against FIN-1, a Wuhan-like strain from January 2020 using VE6 cells both with and without TMPRSS2 expression to a D614G isolate C1P1 using VE6 cells expressing TMPRSS2 (VE6T). The NAb titers in a microneutralization test (MNT) were significantly higher with the non-VE6-adapted C1P1-virus strain and VE6T cells (Geometric mean titer (GMT) 133) than with the VE6-adapted FIN-1 in either cell line (GMT 53 with VE6-cells and 66 with VE6T cells) (*p* < 0.001). There were no differences in NAb titers against VE6-adapted virus strain in VE6T compared to VE6 cells (*p* = 0.685) (Table S3). The overall higher titers of the tested samples on VE6T cells with the C1P1 strain and the differences between the cell culture systems suggest that comparisons between variant strains should preferably be done using cell lines with relevant molecules affecting entry and representative low-passage clinical isolates of the strains. Consequently, further comparisons to variant strains were performed with the C1P1 and VE6T cells.

### 3.2. Overall NAb Titers against C1P1 Compared to Variant Strains

**Figure 1:**
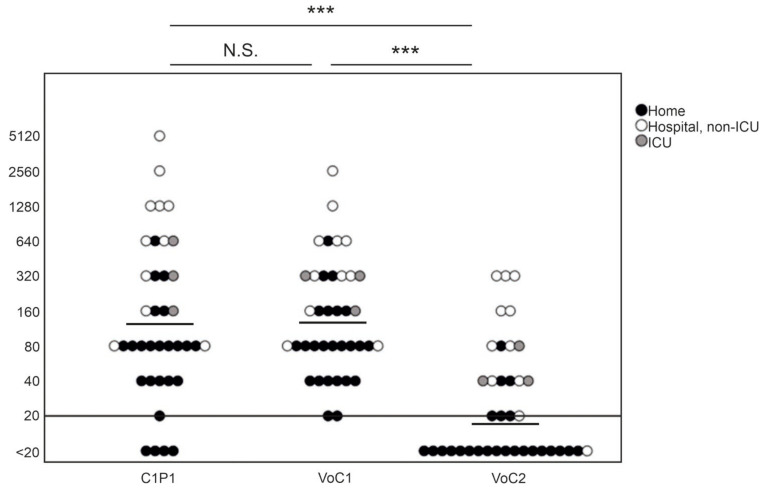
Comparison of neutralizing antibody titers with geometric mean lines against different SARS-CoV-2 strains. Comparison of C1P1, VoC1, and VoC2 titers with individual data points indicated in the picture. Titers are expressed in logarithmic scale (Log_2_) and LOD has been marked with a horizontal line. Statistical significances are indicated with *** (*p* < 0.001) and N.S. (*p* > 0.05).

NAb titers against C1P1 were compared to VoC1 and VoC2 using VE6T cells (Figure 1, Table S3). The GMT was 141 with the VoC1 strain, and 17 with the VoC2 strain, as compared to 133 with the C1P1. The titers were significantly lower, with an approximately 8-fold lower GMT against VoC2 as compared to both VoC1 and C1P1 (*p* < 0.001). Titers of four samples remained below the first tested dilution (<20) with the C1P1 strain, 19 with the VoC2 strain, and none with the VoC1 strain. There was no statistically significant difference between VoC1 and C1P1 (*p* = 1.000).

### 3.3. Comparison of IgG and NAb Titers

**Figure 2:**
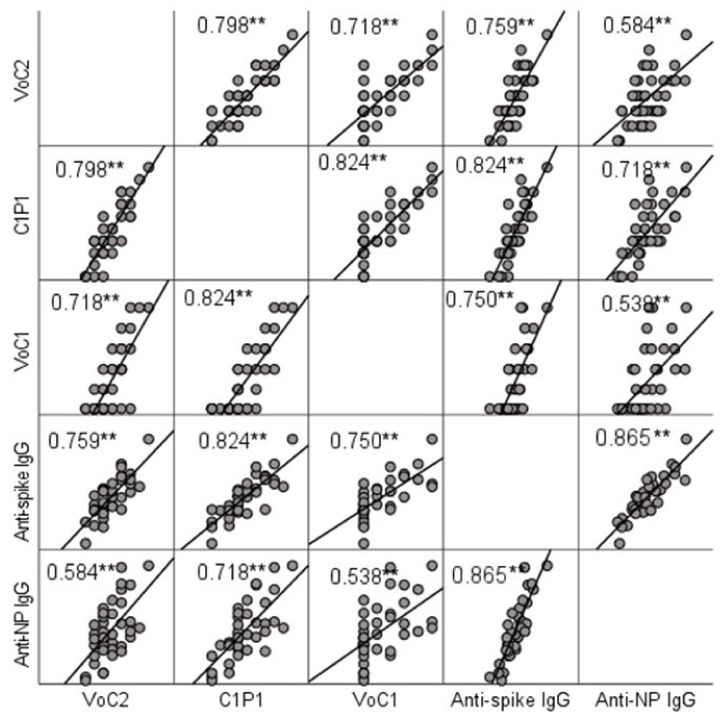
Correlation between NAb titers and IgG titers determined by ELISA testing. Scatter matrix comparing NAb titers with 3 virus strains and IgG titers with spike protein and nucleoprotein. Spearman’s rho-values between NAb titers with each virus strain and anti-spike and anti-NP IgGs are included in the picture, and significant values at level 0.01 (2-tailed) are marked with **.

NAb titers were compared to IgG titers determined by ELISA. A significant positive correlation (*p* < 0.001) with Spearman’s rho-values ranging between 0.584 (anti-NP and VoC2) and 0.824 (anti-spike and C1P1) was found between MNT and ELISA result in all cases (Figure 2, Table S3). Anti-spike-IgG and anti-nucleoprotein-IgG titers did not show a significant difference (*p* = 0.960). 

### 3.4. NAb Titers in Relation to Time from the Disease and Disease Severity

**Figure 3:**
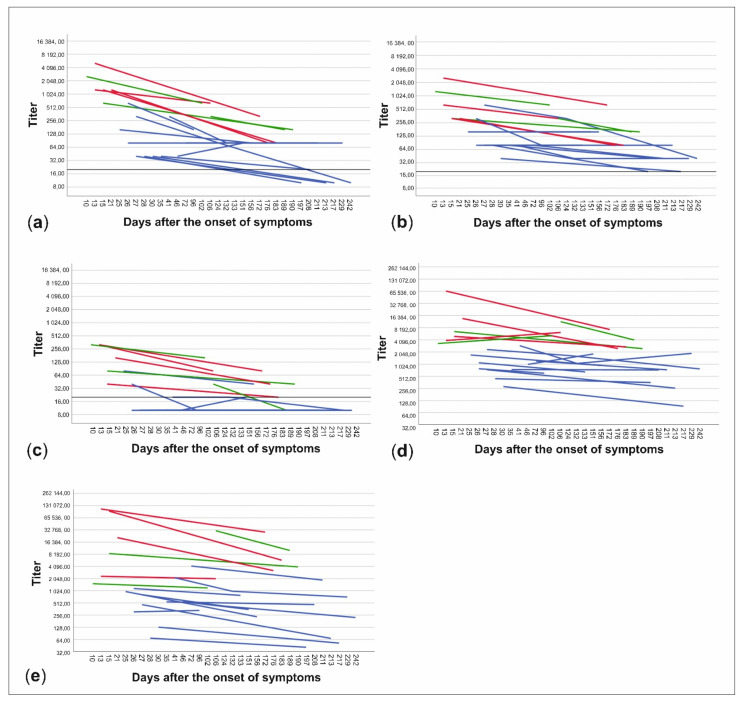
Changes in neutralizing antibody titers and anti-spike- and anti-NP-IgG titers between samples of each patient. Titers are expressed as neutralizing antibody titers for C1P1 (**a**), VoC1 (**b**), and VoC2 (**c**) as well as end-point titers for anti-spike- (**d**) and anti-NP-IgG (**e**) antibodies. Patients treated at home are shown in blue and patients treated in the hospital are shown in red (non-ICU) and green (ICU). Titers are depicted in logarithmic scale (Log2), LOD has been marked with a horizontal line, and titers below LOD in A-C have been set to ten.

The patient data were further divided into subgroups based on whether the patients had been treated at home or had needed hospital care and on the other hand, whether the time from onset of symptoms to date of serum sampling was less than or more than 150 days. NAb titers to all three virus strains and anti-spike- and anti-NP-IgG ELISA titers were higher in patients treated in hospital than in those treated at home (*p* < 0.001) (Figure 3, Table 1). Titers were also higher in the sera drawn less than 150 days from the onset of illness in all cases, but the difference was statistically significant only with C1P1 (*p* = 0.007) and VoC1 (*p* = 0.012) (Table 1, Figure 3, and Table S3). Using VoC2, 5/7 samples taken between days 150–200 were NAb-positive but none of the 6 samples taken after 200 days had detectable levels of NAbs, whereas with C1P1 and VoC1, a large proportion of the patients still had detectable levels of NAbs 200 days after the onset of disease. 

Slopes of fit lines between paired samples of individual patients were used to compare the rates of NAb titer decrease between virus strains. The average slope value was slightly more negative (i.e., the descending slope was steeper) with C1P1 (−1.1 × 10^−2^, SD 1.1 × 10^−2^) than VoC1 (−9.41 × 10^−3^, SD 9.02 × 10^−3^), or VoC2 (−9.90 × 10^−3^ SD 4.84 × 10^−3^), but differences were not statistically significant with Friedman’s analysis of variance by ranks test (*p* = 0.186) or in pairwise comparisons with the Wilcoxon signed rank test (Table S3), suggesting that the waning of the NAb titers occurred at similar pace towards the different variants.

## 4. Discussion

The emergence of new VoC strains of SARS-CoV-2 requires active research to understand their phenotypic and antigenic properties of relevance for the pandemic and its control. This study contributes to the gathering of information on the neutralizing antibody kinetics of previously infected individuals towards clinical isolates representing different strains of SARS-CoV-2. The patients from which the paired samples in this study were collected with high likelihood experienced D614G infection in spring 2020 [25]. These strains are represented by the C1P1 strain, whereas now (April 2021) in Finland the B.1.1.7 strain represents ca. 70% and B.1.351 10–20% of circulating strains. This is why these strains isolated from Finnish patients were selected for MNT analyses.

The obtained results are in line with previous studies reporting lowered NAb levels against the VoC2 (B.1.351) variant when compared to VoC1 (B.1.1.7) or the older dominant strains, including studies in vaccinees [5,8,11,13,26,27]. It should be noted that a large proportion of VoC2 titers were under the detection limit of 20 and were set to value of 10 for calculations, which means that statistical values of VoC2 are not precise. However, the significance of the difference between strains can be judged to be reliable and the relatively high value of 10 was used to avoid reporting false significance. The weak NAb immunity of the first wave recoverees towards VoC2 suggest that the latter lineage poses a public health threat for early re-emergence of SARS-CoV2 in previously infected populations. The results provide additional evidence justifying measures to control the spread of VoC2 and other lineages with immune escape properties. 

The neutralizing response towards the contemporary and VoC1 strains, although waning, was shown to last for months. No differences were detected in the relative rate in which the titer towards each virus strain decreased within this dataset. One should note, however, that due to the large number of samples excluded from this comparison because of the titers below the detection limit, the sample size in the analysis was small. Furthermore, the decrease in NAb response might not be linear in the 2-log scale as was assumed here. In any case, none of the samples taken over 200 days after disease onset showed detectable levels of NAbs against VoC2, which can be explained by the significantly lower initial levels. This indicates that protection provided by NAb response from an infection with strains circulating in 2020 does not last as long against VoC2 as it does against C1P1 and VoC1. 

ELISA and NAb titers correlated well and this suggests that ELISA could serve as a rapid and convenient screening method for identifying high NAb level patients. As in other studies [28,29,30,31], significantly higher antibody responses in both ELISA and NAb tests were measured in patients who needed hospital care as compared to those requiring only outpatient care. The individual NAb responses also showed the presence of occasional “pan-reactive” recoverees with high NAb titers to all variants (Table S1), which could serve as potential donors for e.g., memory B cells for cloning antibodies for therapeutic purposes. 

Although previous studies have shown that in general the different types of SARS-CoV-2 neutralization assays have good concordance [32,33], comparisons of different assays are needed for evaluating the properties of different circulating virus strains that represent naturally occurring sets of mutations. The current understanding of SARS-CoV-2 NAb kinetics is based on results obtained using various techniques, cell lines, and virus strains [6]. Our observation that the TMPRSS2-expressing cells made a VE6 cell-based microneutralization test more sensitive translates to an overall better longevity of the antibody response, and could imply that the results obtained using different assay protocols providing heterologous entry molecules for the virus may vary, calling for further standardization of the NAb assays [5,34]. 

In conclusion, our results support that the strains largely circulating in 2020 provide sustained antibody-mediated protection towards the contemporary strains as well as the B.1.1.7 variant of concern rapidly spreading in e.g., Europe and the United States, but poorly against the B.1.351 variant, explaining its potential for surge in previously infected populations and highlighting the need for control measures to prevent this variant from further spread [35,36,37].

## Figures and Tables

**Table 1:** Geometric mean titers and *p*-values when the data is divided into subgroups based on the time after the onset of symptoms and disease severity.

	Treatment Place			Days After Onset		
	Hospital	Home	*p*-Value	0–150	150–300	*p*-Value
C1P1 NAb	526	60	0.000	241	49	0.007
VoC1 NAb	352	82	0.000	202	76	0.012
VoC2 NAb	76	13	0.000	32	17	0.247
anti-NP IgG	8599	413	0.000	1663	792	0.301
anti-S IgG	5997	820	0.000	2165	1144	0.235

## Data Availability

The data presented in this study are available in the article and in its online Supplementary Materials.

## Supplementary Materials

The following are available online at https://www.mdpi.com/article/10.3390/v13060996/s1, Table S1: Sample information and results. Table S2: Mutations of virus strains used in the study compared to strain Wuhan Hu-1. Table S3: Statistical tests and parameters.
